# Regions of white matter abnormalities in the arcuate fasciculus in veterans with anger and aggression problems

**DOI:** 10.1007/s00429-019-02016-2

**Published:** 2019-12-27

**Authors:** Szabolcs David, Lieke Heesink, Elbert Geuze, Thomas Gladwin, Jack van Honk, Rolf Kleber, Alexander Leemans

**Affiliations:** 1 Image Sciences Institute, University Medical Center Utrecht, Utrecht, The Netherlands; 2 Department of Psychiatry, University Medical Center Utrecht, Utrecht, The Netherlands; 3 Research Center Military Mental Health Care, Utrecht, The Netherlands; 4 Department of Psychology and Counselling, University of Chichester, Chichester, UK; 5 Department of Psychology, Utrecht University, Utrecht, The Netherlands; 6 Department of Psychiatry and Institute of Infectious Diseases and Molecular Medicine (IDM), University of Cape Town, Cape Town, South Africa; 7 ARQ National Psychotrauma Centre, Diemen, The Netherlands

**Keywords:** Aggression, Anger, Veterans, Diffusion tensor imaging, Uncinate fasciculus, Arcuate fasciculus

## Abstract

Aggression after military deployment is a common occurrence in veterans. Neurobiological research has shown that aggression is associated with a dysfunction in a network connecting brain regions implicated in threat processing and emotion regulation. However, aggression may also be related to deficits in networks underlying communication and social cognition. The uncinate and arcuate fasciculi are integral to these networks, thus studying potential abnormalities in these white matter connections can further our understanding of anger and aggression problems in military veterans. Here, we use diffusion tensor imaging tractography to investigate white matter microstructural properties of the uncinate fasciculus and the arcuate fasciculus in veterans with and without anger and aggression problems. A control tract, the parahippocampal cingulum was also included in the analyses. More specifically, fractional anisotropy (FA) estimates are derived along the trajectory from all fiber pathways and compared between both groups. No between-group FA differences are observed for the uncinate fasciculus and the cingulum, however parts of the arcuate fasciculus show a significantly lower FA in the group of veterans with aggression and anger problems. Our data suggest that abnormalities in arcuate fasciculus white matter connectivity that are related to self-regulation may play an important role in the etiology of anger and aggression in military veterans.

**Electronic supplementary material:**

The online version of this article (10.1007/s00429-019-02016-2) contains supplementary material, which is available to authorized users.

## Introduction

Anger and aggression problems are frequently reported in veterans after military deployment (Elbogen et al. 2013; Reijnen et al. 2015; Shea et al. 2018). In a sample of 1090 USA military veterans, 9% endorsed engaging in severe violence and 26% in other physical aggression in the previous year of the study (Elbogen et al. 2014). A meta-analysis of 17 studies on the prevalence of aggressive and violent behavior among the military, aggressive behavior was present with estimates of 10% for physical assault and 29% for all types of physical aggression in the last month. Rates were increased among combat-exposed personnel (MacManus et al. 2015) from the United States and the United Kingdom following deployment to Iraq and/or Afghanistan. In the National Vietnam Veterans Readjustment Study (NVVRS), 33% of male USA veterans with current post-traumatic stress disorder (PTSD) reported intimate partner aggression in the previous year (Jordan et al. 1992). These problems hardly diminished over time (Heesink et al. 2015) and often remained even after treatment (Shin et al. 2012). The findings underline the importance of research into the etiology of anger and aggression to improve treatment strategies.

Anger and aggressive behavior are related to a network of emotion processing brain regions, the amygdala, anterior cingulate, hypothalamus, and brain stem; as well as inhibitory and value processing prefrontal regions, the ventromedial, and orbitofrontal prefrontal cortex (Blair 2016; Waller et al. 2017). Functional connectivity studies in populations with clear indications of anger and aggression have found evidence of reduced inhibitory interactions between frontal areas and the amygdala (Best et al. 2002; Coccaro et al. 2007; Varkevisser et al. 2017). These functional differences involving emotional processing, cognitive control, and attention might involve structural abnormalities in white matter connectivity. Diffusion tensor imaging (DTI) studies in impulsive aggression are scarce, but a tract that may be of interest given previous work is the uncinate fasciculus (UF) (Dailey et al. 2018). The UF connects the frontal lobe and temporal pole structures including the amygdala (Catani et al. 2002; Schmahmann et al. 2007) and is related to the use of social–emotional information in decision-making (Von Der Heide et al. 2013). A recently published systematic review showed that in adults with antisocial disorder, the diffusion characteristics of the UF are altered (Waller et al. 2017). Furthermore, white matter abnormalities in the UF have been linked to aggressive behavior in non-clinical populations of adults (Peper et al. 2015).

The arcuate fasciculus (AF) is also of interest to the current work. The AF connects frontal, temporal, and parietal regions related to social cognition (Bernhardt et al. 2014). To the best of our knowledge, this tract has not yet been investigated in an aggression focused DTI study, but known associations between the AF and various psychological processes suggest that it could be relevant to aggression as well. The AF is related to emotion regulation (Sun et al. 2017), mentalizing (Nakajima et al. 2018), language (Kamali et al. 2014; Schomers et al. 2017), and, of particular interest, the social use of language (Catani and Dawson 2016). Deficits in language are known to be a risk factor in anger and aggression (Miller et al. 2008; Teten et al. 2010). Lower FA values in the AF have also previously been linked to mood disorders (Spitz et al. 2017).

To conceptually verify our results, we included analyses of a subdivision of the cingulum as a control tract in which differences between healthy and pathological aggression populations are not expected. The cingulum is a multi-component, complex fiber system, which can be divided into distinct subdivisions (Jones et al. 2013; Heilbronner and Haber 2014). The current status of the subdivisions differentiates the following parts going from the most frontal toward dorsal and temporal components: subgenual, anterior cingulate, midcingulate, retrosplenial, and parahippocampal portions. The subdivisions are based on studies investigating quantitative measures, for example DTI metrics, of the tract parts (Concha et al. 2005; Jones et al. 2013; Lin et al. 2014; Metzler-Baddeley et al. 2017). Among the subdivisions, the parahippocampal cingulum was selected, which is running within the parahippocampal gyrus or Broca Area (BA) 34 and 28, retrosplenial cingulate gyrus (BA 26, 19, and 30) (Thiebaut de Schotten et al. 2012; Mandonnet et al. 2018), connecting the posterior cingulate cortex and medial temporal lobe. The parahippocampal cingulum has been linked to memory and visuospatial working memory in the general population (Zahr et al. 2009; Bubb et al. 2018) and in patient groups with temporal lobe epilepsy (TLE) (Winston et al. 2013), velocardiofacial syndrome (VCFS), also called 22q11.2 deletion syndrome (Kates et al. 2007) and autism spectrum disorder (ASD) (Chien et al. 2016).

The aim of the current study is to determine whether tissue microstructure of the AF or the UF as assessed with the FA is related to anger and aggression. Tract pathways were reconstructed using fiber tractography, and comparisons of the FA of whole tracts and segments of tracts were compared between veterans with anger and aggression and a control group of veterans who had also been in combat, but did not suffer from anger and aggression problems.

## Methods

### Participants

This study included 29 male veterans with anger and aggression (Aggression group) and 30 control veterans (Control group). Participants in the Aggression group were recruited via their psychologists/psychiatrists at one of the outpatient clinics of the Military Mental Health Care Institute or via advertisements in the waiting room and newsletters for veterans. Control participants were recruited by advertisements or had participated in previous studies. The two groups were matched for number of deployments, education, and age. Inclusion criteria for the Aggression group were based on the four research criteria for impulsive aggression described by Coccaro (2012): (1) verbal or physical aggression towards other people occurring at least twice weekly on average for 1 month; or three episodes of physical assault over a 1 year period; (2) the degree of aggressiveness is grossly out of proportion; (3) the aggressive behavior is impulsive (not premeditated); (4) the aggressive behavior causes either distress in the individual or impairment in occupational or interpersonal functioning (Coccaro, 2012). Inclusion criteria for the Control group were (1) no current DSM-IV diagnosis; (2) no history of pathologic aggressive behavior.

### Interview and questionnaires

The Dutch version of the International Neuropsychiatric Interview (MINI) was used to screen for the presence of comorbid psychiatric disorders (Overbeek et al. 1999). The complete MINI was administered. In this interview, the following current or life-time disorders were screened: depressive disorder, dysthymia, suicidal risk, (hypo)manic disorder, panic disorder, anxiety disorder, agoraphobia, social phobia, obsessive compulsive disorder, PTSD, alcohol or drug dependence and/or abuse, psychotic disorders, anorexia nervosa, bulimia nervosa, generalized anxiety disorder, antisocial personality disorder, somatization disorder, hypochondria, body dysmorphic disorder, pain disorder, attention deficit hyperactivity disorder (ADHD), and adjustment disorder.

To measure anger and aggression, two questionnaires were administered. First, the Dutch version of the State-Trait Anger Expression Inventory-revised (STAXI-2; Hovens et al. 2015, Spielberger 1999) was used. The STAXI-2 consists of 57 items on a 4-point Likert scale and is divided into two subscales: State Anger and Trait Anger. Furthermore, the Dutch translation of the Buss-Perry Aggression Questionnaire (AQ) (Buss and Perry 1992; Meesters et al. 1996) was administered. The AQ consists of 29 items on a 5-point Likert scale and is divided into four subscales: Physical Aggression, Verbal Aggression, Anger, and Hostility.

### Data acquisition

All data sets were acquired using a 3 T MRI scanner (Philips Medical System, Best, The Netherlands). Two diffusion MRI scans were collected; one with posterior–anterior (PA) and one with anterior–posterior (AP) phase-encoding directions, each with one non-diffusion-weighted image (*b* = 0 s/mm^2^) and 30 diffusion-weighted images (*b* = 1000 s/mm^2^), where the distribution of the diffusion-weighted gradients was based on work by Jones et al. (Jones et al. 1999). The acquisition settings were: TR = 7057 ms, TE = 68 ms, voxel size = 1.875 × 1.875 × 2 mm^3^, 75 slices, and slice thickness = 2 mm without gap, FOV = 240 × 240 mm^2^, matrix size = 128 × 128. Details of the T1 weighted anatomical scan: TR = 10 ms, TE = 4.6 ms, flip angle = 8°, voxel size = 0.8 × 0.8 × 0.8 mm^3^, FOV = 240 × 240 mm^2^, matrix size = 304 × 299.

### Data processing

The diffusion MRI data sets were processed using *FSL* (v5.0.9) (Jenkinson et al. 2012) and *ExploreDTI* (v4.8.6) (Leemans et al. 2009). First, susceptibility distortions were estimated with *topup* (Andersson et al. 2003) which were an input for *eddy* (Andersson and Sotiropoulos 2016) to correct for motion, geometrical distortions, and rotation of the diffusion gradient orientations (Leemans and Jones 2009). Other settings of *eddy* were left at default values. Robust extraction of brain tissue was executed with BET (Smith 2002). DTI estimation was performed using REKINDLE (Tax et al. 2015). Whole brain tractography was performed with the following parameter settings: seed FA threshold = 0.2; angle threshold = 30° (Basser et al. 2000).

**Fig. 1:**
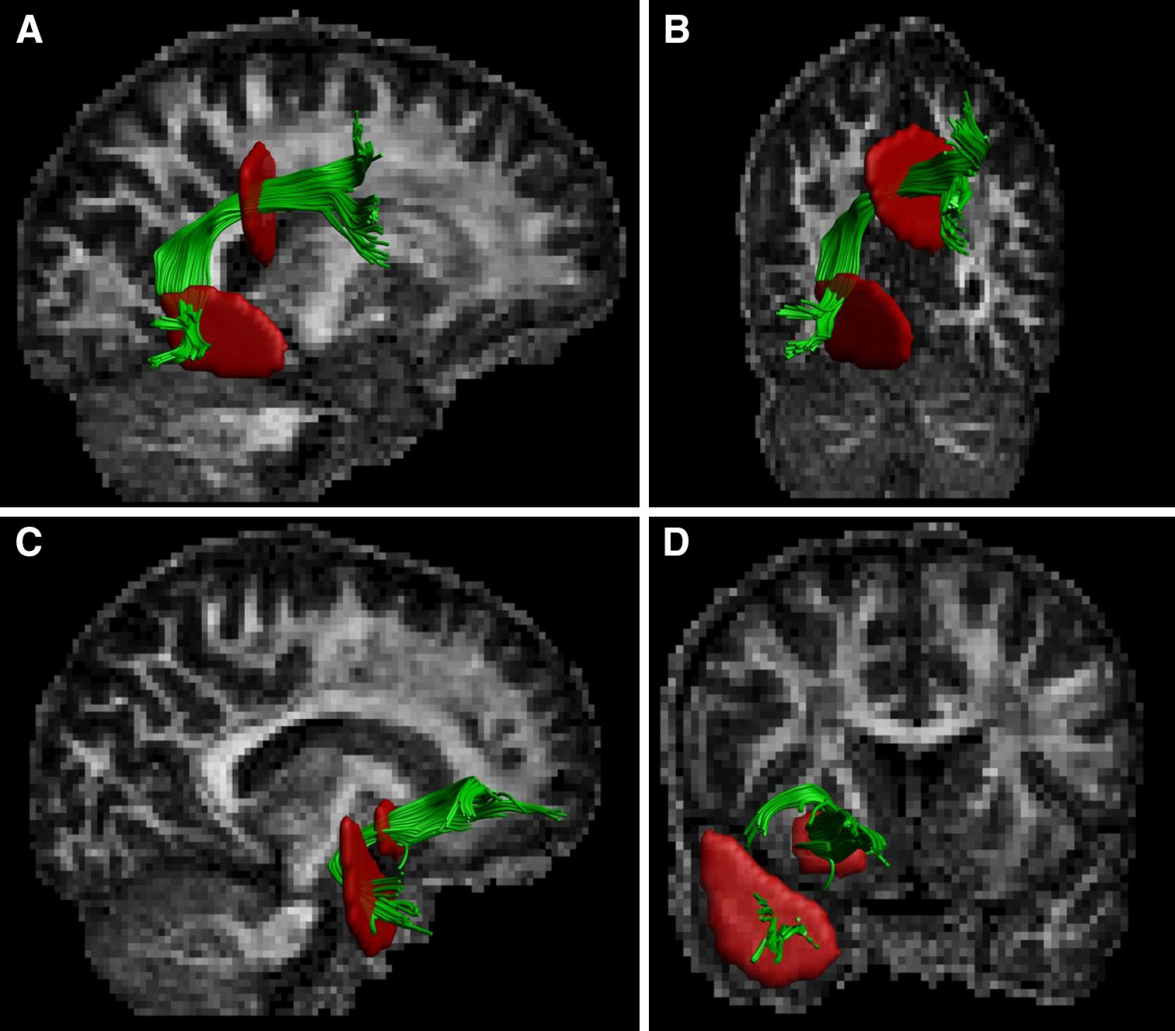
Configurations of regions of interest (ROIs) that are used for tractography to segment the right arcuate (sagittal: A and coronal: B) and the right uncinate (sagittal: C and coronal: D) fasciculi in a representative subject. The ROIs are shown in red and the tracts in green with the fractional anisotropy as the background map

Reconstruction of both AFs (left and right; we did not have any priori hypotheses concerning laterality) were performed by placing two Boolean “AND” regions of interest (ROIs) (Conturo et al. 1999; Catani et al. 2002; Wakana et al. 2007). The first ROI was placed on the most posterior coronal slice showing the fornix on the midline to include the pathways laterally to the corona radiata trajectories running towards the frontal lobe. The second ROI was placed on a sagittal slice to include the pathways going towards the temporal lobe. Figure 1a, b shows the positions of the ROIs for the reconstruction of the AF.

Reconstruction of the UF (left and right) was performed by placing two Boolean “AND” ROIs on the most posterior coronal slice, where the temporal and frontal lobes were separated (Conturo et al. 1999; Catani et al. 2002; Wakana et al. 2007). The first ROI included the entire temporal lobe, and the second ROI included all pathways running towards the frontal lobe. Obvious artifacts (lines running towards the occipital lobe or lines over the midline) were removed by “NOT” ROIs. Figure 1c, d shows the positions of the ROIs for the reconstruction of the UF.

Reconstruction of the cingulum (left and right) was performed by placing two Boolean “AND” ROIs in the coronal plane. The first is at the middle of the splenium of the corpus callosum (CC), while the second is middle of genu of CC (Conturo et al. 1999; Catani et al. 2002; Wakana et al. 2007). An additional “NOT” ROI was placed in the midsagittal plane to exclude interhemispheric fibers, which are non-plausible for the cingulum. Supplementary Fig. 1 shows the position of the “AND” ROIs for the parahippocampal cingulum.

### Statistical analyses

The mean FA values over the whole tracts were computed and compared between groups. Furthermore, a segment-wise analysis was performed to investigate the properties of the tract pathways along the trajectory as described previously (Colby et al. 2012; Szczepankiewicz et al. 2013; Reijmer et al. 2013; O’Hanlon et al. 2015). For all the three bundles, three positions at the ends of the pathways were excluded from the analyses to minimize partial volume effects (Vos et al. 2011). FA values over the length of the left and right UF, AF, and cingulum were compared between groups using the following two-step approach. First, between-group t tests were performed for each 2 mm segment along the tract separately. Second, permutation tests were performed to test whether the length of sequences of consecutive nominally significant segments was above chance level. The null-hypothesis distribution of this nominally significant sequence length was determined using permutation tests as used in the previous studies (Gladwin et al. 2016). Permutation tests allow a simple and valid approach to estimate distributions involving non-independent tests (Nichols and Holmes 2002; Eklund et al. 2016), such as those for different positions in the current analyses. The permutation procedure consisted of randomizing group assignment and was done for 10,000 permutations. From these permutations, a null-hypothesis distribution of the longest sequence of consecutive nominally significant segments over the whole tract was computed and used to test observed sequence lengths. Using this approach, false-positive rate is controlled for over the whole fiber trajectory. This approach may be more sensitive to localized abnormalities than using the mean FA over the whole tract.

## Results

### Demographics

The groups did not differ on age, education, number of deployments, and time since last deployment (all *p*’s > 0.10). As expected, the Aggression group showed significantly higher scores on all anger and aggression measures compared to the Control group. Table 1 shows statistics of the demographic data and questionnaire data.

**Table 1:** Demographics of the anger group and the control group

	Anger group (*N* = 29)	Control group (*N* = 30)	Statistics
	Mean (SD)	Mean (SD)	
Age (years)	36.28 (6.31)	34.53 (7.59)	*t* (57) = 0.96, *p* = 0.34
Education	4.21 (0.62)	4.2 (0.81)	*t* (57) = 0.04, *p* = 0.97
Number of deployments	2.07 (1.16)	2.37 (1.25)	*t* (57) = − 0.95, *p* = 0.35
*STAXI-2*			
*State Anger*	24.07 (11.30)	15.20 (0.76)	*t* (57) = 4.29, *p* < 0.001
*Trait Anger*	23.03 (7.01)	12.13 (2.47)	*t* (57) = 8.02, *p* < 0.001
*Aggression Questionnaire*			
Physical aggression	30.07 (7.48)	18.47 (4.55)	*t* (57) = 7.22, *p* < 0.001
Verbal aggression	15.66 (3.97)	11.3 (1.54)	*t* (57) = 5.60, *p* < 0.001
Anger	24.48 (5.34)	11.17 (2.49)	*t* (57) = 12.35, *p* < 0.001
Hostility	24.24 (7.00)	11.87 (3.41)	*t* (57) = 8.68, *p* < 0.001

*SD* standard deviation, *STAXI-2* State-Trait Aggression Inventory-revised

### Mean FA values per tract

Reconstruction of the left and right UF was possible in all participants; it failed for the left AF in six participants and for the right AF in one participant; and reconstruction of the left and right cingulum was possible in all participants. Supplementary Fig. 2 shows the tracking results of ten representative subjects for the UF and AF. Between the groups, there were no significant differences in mean FA values for each fiber bundle (UF right: *t*(57) = 0.120, *p* = 0.91; UF left: *t*(57) = 0.193, *p* = 0.85; AF right: *t*(56) = 1.123, *p* = 0.27; AF left: *t*(51) = 0.934, *p* = 0.36; cingulum left bundle: *t*(57) = 0.826, *p* = 0.412; cingulum right: *t*(57) = − 0.328, *p* = 0.744.

### Along-tract analyses

#### Uncinate fasciculus

**Fig. 2:**
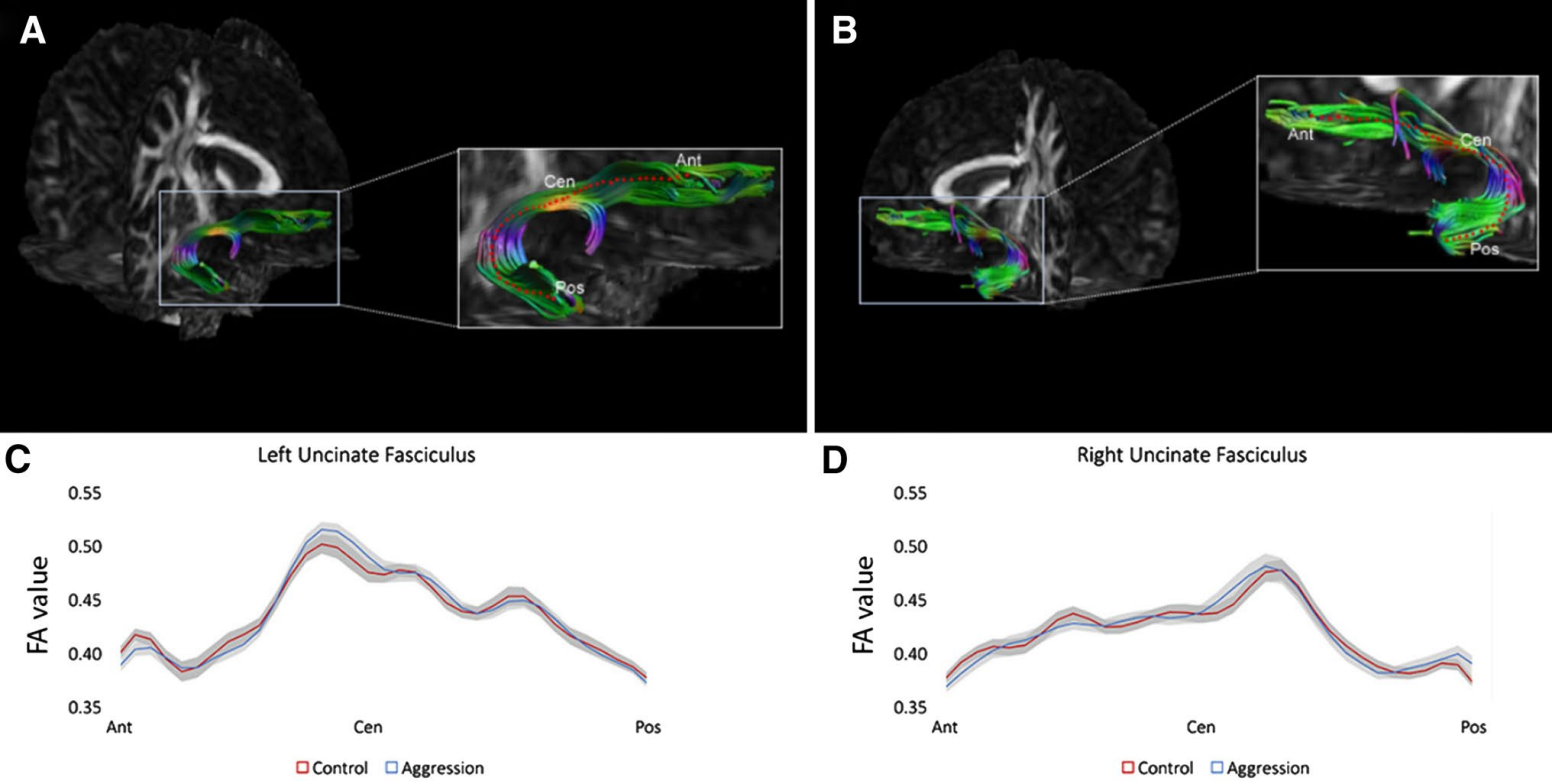
Schematic overview of the along-tract analysis for the uncinate fasciculi (UF). An example of the left and right UF is shown in A and B. C and D show the FA values along the left and right UF, respectively

between-group *t* tests showed one nominally significant difference along the right UF tract pathway (*t*(57) = 2.05, *p* = 0.045, uncorrected). However, this was not sufficient to achieve whole-tract significance using the permutation test. The left UF showed no significant differences along the tract pathway (all *p* values > 0.20). The FA values along the right and left UF are depicted in Fig. 2.

#### Arcuate fasciculus

**Fig. 3:**
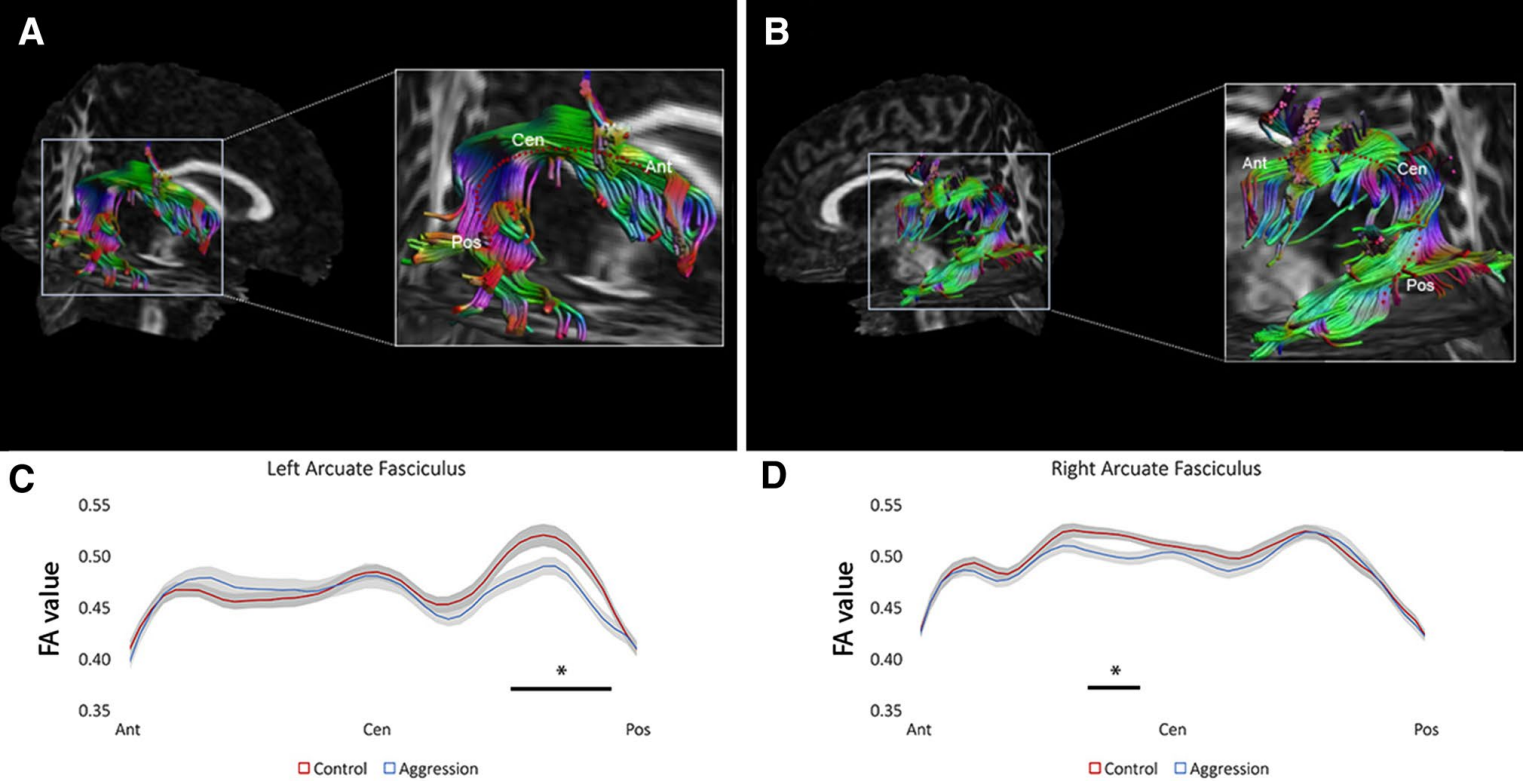
Schematic overview of the along-tract analysis in the arcuate fasciculi (AF). An example of the left and right AF is shown in A and B. C and D show the FA values along the left and right AF, respectively, where the black lines highlight significant differences between the two groups

Figure 3 shows the between group t-test results for both the right and left AF tracts. Significant differences on the left AF were found at 66 mm (*t*(51) = 2.196,* p* = 0.03), 68 mm (*t*(51) = 2.301, *p* = 0.03), 70 mm (*t*(51) = 2.124, *p* = 0.04), 74 mm (*t*(51) = 2.107, *p* = 0.04), 76 mm (*t*(51) = 2.569, *p* < 0.01), 78 mm (*t*(51) = 2.910, *p* = 0.005), and at 80 mm (*t*(51) = 2.615, *p* < 0.01), where the positions are measured from the anterior end of the tracts. Permutation tests showed that this number of consecutive significant points was significant (*p* = 0.019). The FA values along the right and left AF are depicted in Fig. 3. Significant differences on the right AF were found at 32 mm (*t*(56) = 2.170, *p* = 0.03), 34 mm (*t*(56) = 2.536, *p* = 0.01), 36 mm (*t*(56) = 2.782, *p* = 0.01), 38 mm (*t*(56) = 2.647, *p* = 0.01), and at 40 mm (*t*(56) = 2.167, *p* = 0.03). Permutation tests showed that this number of consecutive significant points did not reach the threshold for significance (*p* = 0.091).

#### Cingulum

Along the tract permutation tests showed no significance for both left and right cingulum. The along the tract FA values are depicted in Supplementary Fig. 3.

## Discussion

This study was performed to test whether combat veterans with anger and aggression differ in white matter structure in the UF and AF from combat veterans without anger and aggression. The UF and AF play a role in the regulation of emotion and attention and are, therefore, of interest in anger and aggression. No differences between the two groups were found in the UF, but evidence pointed at lower FA in the AF in the veterans with anger and aggression.

The AF connects the dorsolateral prefrontal cortex with posterior parietal and temporal regions (Makris et al. 2005). Using tract-based spatial statistics, altered white matter microstructure was also reported for the AF in intermittent explosive disorder (IED), a psychological disorder characterized by impulsive aggression (Lee et al. 2016). The finding of lower FA values within the AF for the Aggression group in the current study provides further evidence that white matter organization within this area plays an important role in anger and aggression. The role of the AF is primarily related to cognitive functioning and language (Schomers et al. 2017). Why could this play a role in aggression? First, anger and aggression in veterans have been linked to alexithymia (Teten et al. 2008; Miller et al. 2008), a condition that is characterized by reduced emotional self-awareness and that is associated with lower FA values in AF (Kubota et al. 2012). Second, the link between lower FA values in the AF and mentalizing systems has been reported in autism (Kana et al. 2014). Concerning the role of AF in language, it plays a role in complex comprehension, social communication, and higher semantic processing in particular (Catani and Dawson 2016). Taken together, this suggests that the relationship between reduced AF connectivity and aggression could be mediated via deficits in emotional self-awareness, interpretation, and expression, as these AF-related processes also play a role in the ability to appropriately regulate emotions and aggression (Scheier et al. 1974; Cole et al. 1994; Cohn et al. 2010; Mohammadiarya et al. 2012; Locke et al. 2015; Hawes et al. 2016; Wegrzyn et al. 2017). Future research appears warranted to follow this line of studies to uncover these relationships in more detail.

No differences in FA values along the UF were found in the current study. In a previous study in healthy individuals, no link was found between UF microstructure and trait aggressiveness (Beyer et al. 2014). Furthermore, in a study with IED patients, no differences in white matter in brain areas corresponding to the UF were found as well (Lee et al. 2016). Altered UF microstructure is, however, related to antisocial behavior (Waller et al. 2017) and especially psychopathy (Craig et al. 2009). In this context, disconnection studies of Phineas Gage revealed that his aggressive behavior was related to the damage of the UF (Van Horn et al. 2012; Thiebaut de Schotten et al. 2015). The relationship between UF organization and aggressive behavior might depend on whether aggression is antisocial or instrumental in nature. The current population of veterans is characterized by impulsive aggression rather than psychopathic behavior, potentially explaining the absence of effects for the UF.

Research into the etiology of anger and aggression in military veterans needs to be extended beyond fronto-limbic dysfunction. Brain networks involved in attention and executive functioning, including prefrontal and parietal cortex (Wager and Smith 2003; Van Hecke et al. 2013), may play an important role as well, as shown by abnormal white matter microstructure in parietal regions of the SLF (Karlsgodt et al. 2015). The current study also shows that analysis of a diffusion measure of interest along the tract, instead of one global estimate per tract, is valuable, as this kind of analysis can be more sensitive in detecting subtle differences in fiber tract microstructure.

The cross-sectional nature of the current study gives no information regarding the question whether the abnormal microstructure of the AF in veterans with anger and aggression is a cause or a consequence of the problems with anger and aggression during deployment. It should also be noted that the underlying mechanism associated with aggression might differ within our sample. Thus, it is possible that the UF and AF tracts may help to explain some aspects of aggressive behavior in some military veterans, but not in others. A limitation of our study is that the sample size does not allow subgroup analysis. In addition, we acknowledge that our cohort was also limited in gender and age, and thus, we cannot claim that our results would generalize to the full population of military veterans, including women and veterans of different age.

An unexpected finding in the current results was the relative difficulty in detecting the right rather than left AF. However, asymmetric properties of the AF are not unprecedented, as lateral differences of the tract have been showed previously (Dubois et al. 2009; Lebel and Beaulieu 2009; Allendorfer et al. 2016; Reynolds et al. 2019). While DTI-based fiber tractography (Mori et al. 1999; Basser et al. 2000) is still the most widely used approach in a clinical setting, there are nowadays more accurate approaches to compute fiber orientations, such as those based on spherical deconvolution approaches (Tournier et al. 2007; Dell’Acqua et al. 2010; Tax et al. 2014; Jeurissen et al. 2019). In regions with crossing fiber configurations, these advanced tractography techniques have been shown to provide more reliable reconstructions of white matter fiber pathways (Jeurissen et al. 2011; De Schotten et al. 2011; Thiebaut de Schotten et al. 2012; Rojkova et al. 2016; Kenney et al. 2017). In this context, scalar measures derived from DTI, such as the mean diffusivity or the FA used in this work, are also non-specific in regions with multiple crossing fiber pathways and are prone to partial volume effects (Vos et al. 2011, 2012; Jeurissen et al. 2013). With higher angular resolution diffusion MRI acquisitions becoming more and more available in the clinical realm, future work may incorporate more direct and tract-specific measures for studying potential white matter abnormalities in military veterans with problems of anger and aggression (Raffelt et al. 2012; Dell’Acqua et al. 2013).

This study contributes to the understanding of the interplay between information processing and microstructural white matter tissue organization in veterans with anger and aggressive behavior. Disturbances in networks involving the AF responsible for social cognition may render individuals more vulnerable to aggression. Knowledge about this underlying neural substrate could ultimately be used to facilitate target interventions of such vulnerabilities.

## Electronic supplementary material

Below is the link to the electronic supplementary material.

Supplementary file1 (DOCX 25841 kb)
