# Supplementary material for: Regions of white matter abnormalities in the arcuate fasciculus in veterans with anger and aggression problems
Source: Brain Struct Funct. 2019 Dec 27;225(4):1401–11. doi: 10.1007/s00429-019-02016-2 (PMC7271041; doi:10.1007/s00429-019-02016-2)
Supplement: Supplementary file 1 — Supplementary file1 (DOCX 25841 kb) [file 429_2019_2016_MOESM1_ESM.docx]

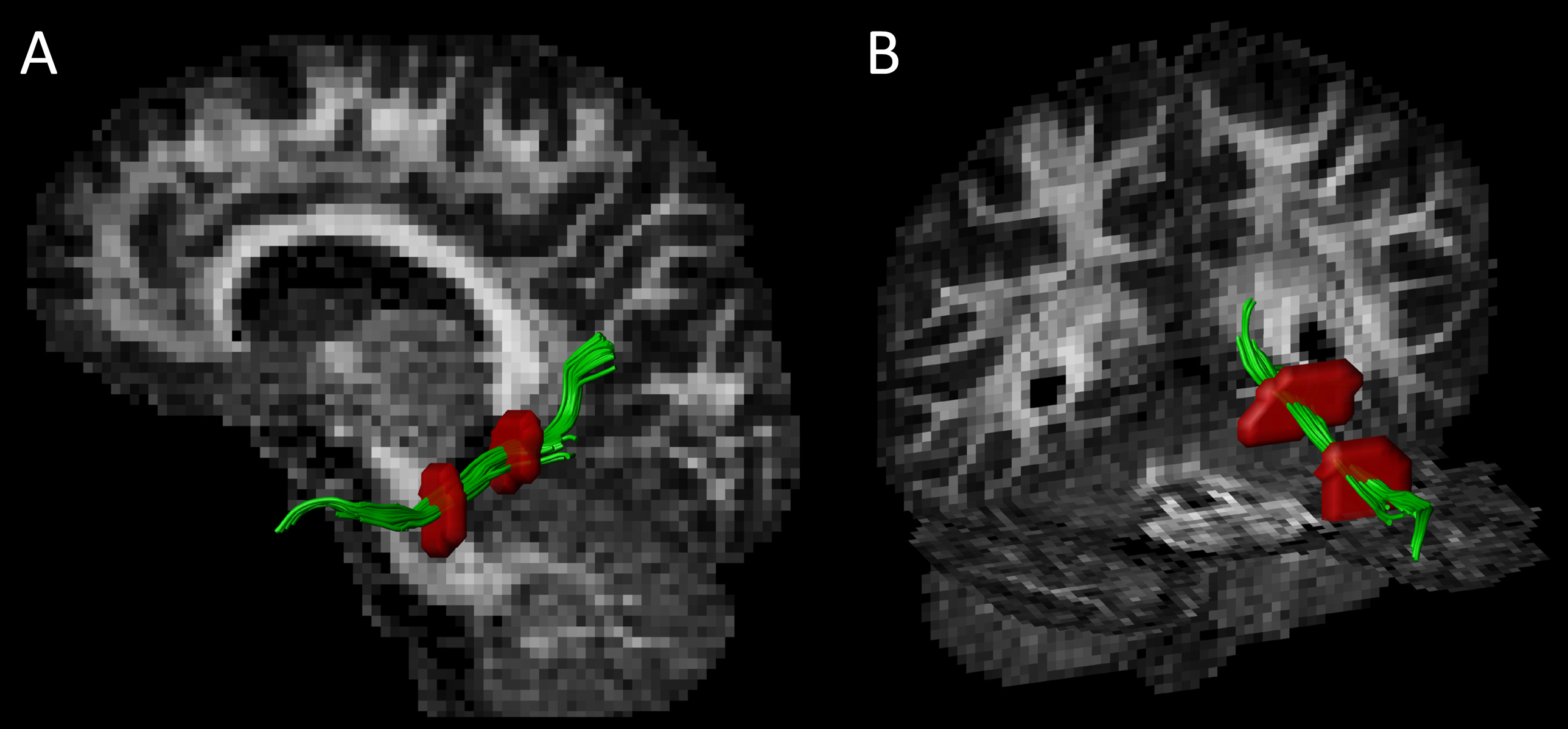


Supplementary Figure 1

Configurations of regions-of-interest (ROIs) that are used for tractography to segment the parahippocampal cingulum (sagittal: A and corona/axial: B) in a representative subject in the left hemispere. The ROIs are shown in red and the tracts in green with the fractional anisotropy as the background map.





Supplementary Figure 2

Reconstructed fiber bundles of both left and right uncinate (UF) and arcuate (AF) fasciculi from ten representative subjects.


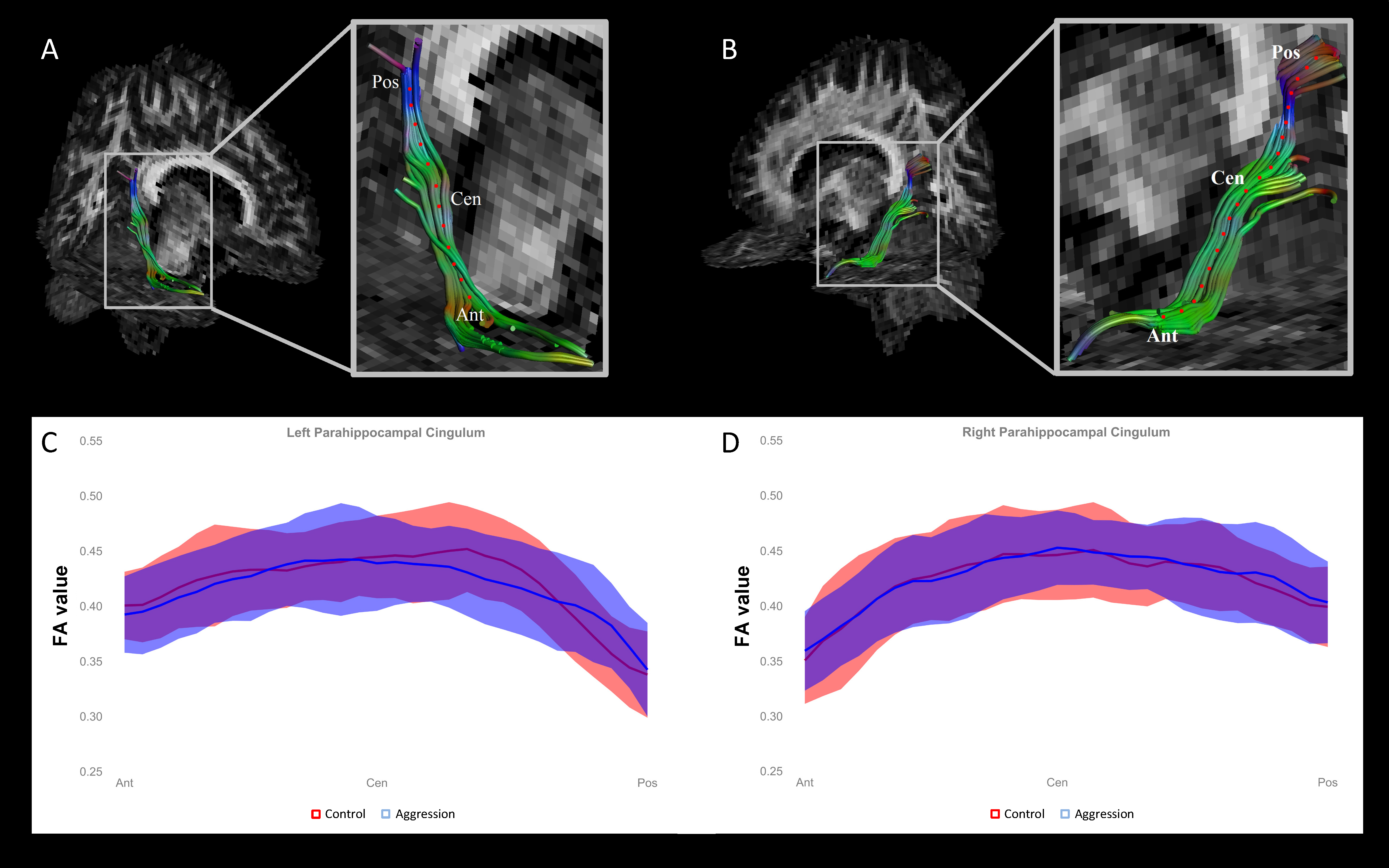


Supplementary Figure 3

Schematic overview of the along-tract analysis for the parahippocampal cingulum. An example of the left and right parahippocampal cingulum is shown in A and B. C and D show the FA values along the left and right tract, respectively.
